# Saccharification and Ethanol Production of Corn Wastes Using a Locally Produced *Trichoderma atroviride* Enzymatic Cocktail

**DOI:** 10.1021/acsomega.5c07764

**Published:** 2025-11-05

**Authors:** Mariana Fornazier Borges, Geraldo Aparecido da Rocha Junior, Daniel Pasquini, Milla Alves Baffi

**Affiliations:** † Federal University of Uberlândia, Chemical Institute (IQ-UFU), Uberlândia, Minas Gerais 38400-902, Brazil; ‡ 28119 Federal University of Uberlândia, Agricultural Sciences Institute (ICIAG-UFU), Uberlândia, Minas Gerais 38405-320, Brazil

## Abstract

This study investigated the efficiency of a homemade enzymatic cocktail from *Trichoderma atroviride* (HTM strain) in the saccharification of corn straw (CS) and corn cob (CC), aiming at cellulosic ethanol production. The blend of cellulases and hemicellulases, obtained through solid-state fermentation, exhibited high levels of β-glucosidase (374.91 U/g) and xylanase (920 U/g). The β-glucosidase from this cocktail presented optimal activity and stability at pH and temperature ranges suitable for lignocellulose bioconversions (4.5–5.0 and 50 °C) and was employed in the saccharification of alkali-pretreated CC and CS (PTCC and PTCS, respectively). The fed-batch hydrolysis, performed with 30 CBU/g (*cellobiase unit*) cellulose and 35% solid (w/v), released the highest concentration of total reducing sugars (TRS) in PTCC (15.86 g/L). When 1% Tween 20 was added to the reaction mixture, a total of 30.37 g/L TRS was detected in PTCC hydrolysate, representing an increase of 91.4% in comparison to the hydrolysis without the surfactant. The alcoholic fermentation of the PTCC hydrolysate resulted in a theoretical conversion yield of 83.48%. These data indicated the feasibility of using *T. atroviride* as a low-cost enzymatic source and the combined strategy of fed-batch mode and surfactant in improving the hydrolytic and fermentative efficiencies.

## Graphical abstract

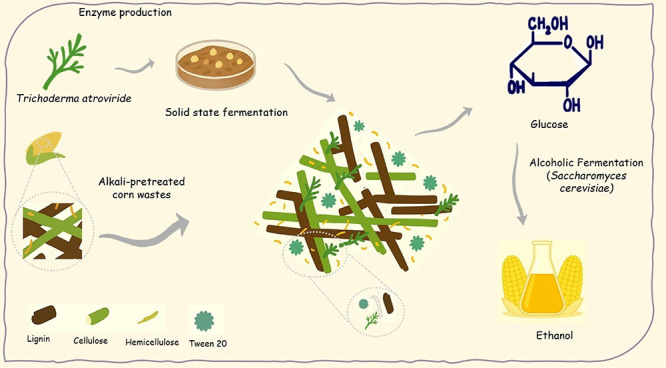

## 1 Introduction

In lignocellulose biorefineries, enzymatic hydrolysis plays a key role in the release of fermentable sugars, which can be converted into second-generation (2G) ethanol and other bioproducts.^1,2^ Saccharifications are mostly performed with commercial cocktails rich in cellulases and hemicellulases, which synergistically act to depolymerize the biomass.^3,4^

Among the cellulases, endoglucanases (EC 3.2.1.4) randomly cleave the β-(1→4) bonds of cellulose in regions of lower crystallinity, exposing reducing and nonreducing chain ends.^5^ These exposed ends become accessible to exoglucanases (EC 3.2.1.91), which act on the crystalline regions and release cellobiose. At last, β-glucosidases (EC 3.2.1.21) convert cellobiose into monomeric glucose.^6,7^ Among the hemicellulases, xylanases (EC 3.2.1.8) and β-xylosidases are responsible for hydrolyzing β-1,4-xylan linkages, resulting in the release of xylose. These enzymes promote the efficient hemicellulose degradation and complement the action of cellulases.^8,9^

To increase the enzyme accessibility to the polysaccharide fractions and improve the hydrolytic efficiency, pretreatments have been applied.^10,11^ Among them, alkaline pretreatments are preferred due to their high efficiency in lignin removal, exposing the cellulosic fraction to degradation.^1^ Although pretreatments play a fundamental role in the enhancement of cellulose availability, the efficacy of hydrolysis still faces economic challenges. The need for high enzyme loadings significantly impacts the process costs.^12,13^ Previous studies have indicated that the saccharification accounts for approximately 20 to 30% of the total cost of ethanol, representing one of the main obstacles to its industrial viability.^13^

On the other hand, the local synthesis of cocktails within the biorefinery emerges as an innovative strategy to reduce the expenses associated with the purchasing of enzymes.^9^ In this sense, the partial replacement of commercial enzymes by locally produced ones has demonstrated significant economic gains.^14^ Among the producers, the genus *Trichoderma* has been reported as one of the most important sources of cellulases and hemicellulases. Traditionally, *T. reesei* has been the most extensively applied due to its high capacity to produce these enzymes.^15,16^ However, few studies have reported the enzymatic biosynthesis by the species *T. atroviride* and their role in saccharifications.^16,17^

In this context, *T. atroviride* emerges as a promising alternative due to its capacity to produce cellulases, especially extracellular β-glucosidase.^18,19^ For that, solid-state fermentation (SSF), using agro-industrial residues, has demonstrated great potential, contributing to the sustainability of bioconversions.^20–22^ This technique efficiently converts these wastes into lignocellulose-degrading enzymes, promoting cost reductions of up to 10-fold.^19^

Another approach to increase the enzymatic digestibility is the addition of surfactants to the reaction medium.^10,20^ The residual lignin in pretreated substrates is a limiting factor in the sugar release due to its interaction with the enzymes, impairing their efficiency.^11^ In this regard, nonionic surfactants, such as Tween 20, can be added to minimize these undesired interactions, enhancing the lignocellulose depolymerization.^23,24^ At last, the enzymatic hydrolysis in fed-batch mode has proven effectiveness in improving biomass degradation.^1,20^ This strategy allows operation at high solid loadings, mitigating the high viscosity and enzyme inhibition, which are common in simple batch processes, increasing the sugar productivity.^1,25^ Thus, the combination of surfactants with the fed-batch strategy can further enhance the hydrolytic efficiency and, consequently, the ethanol yields.^20^ In this context, this study evaluated the production of on-site (hemi)­cellulosic-degrading enzymes by *T. atroviride* via SSF and the effects of temperature and pH on the β-glucosidase activity present in this preparation. The hydrolytic potential of this enzymatic blend was evaluated in alkali-pretreated corn wastes through an integrated strategy of fed-batch mode and the use of Tween 20. Lastly, the 2G ethanol production from the generated hydrolysates was investigated.

## 2 Experimental Section

### 2.1 Microorganism

The fungal strain *T. atroviride* HTM was kindly donated by H.T.M. Comercio e Laboratorios de Corretivos do Solo Ltd.—Biosag (Ituverava, SP, Brazil). This strain was previously identified through gene sequencing using the rRNA SR6R and LR1 primers.^26^ The culture was maintained in Petri dishes with potato dextrose agar (PDA) culture medium at room temperature.

### 2.2 Enzymatic Production by Solid-State Fermentation

Raw wheat bran (WB) was used in SSF as a substrate for fungal development and enzymatic synthesis. WB was purchased from a local cereal market (Uberlandia, MG, Brazil) and washed under running tap water until there were no starch residues. Then, it was dried at room temperature for 48 h, crushed in a blender, sieved to a particle size of approximately 0.6–1.0 cm, and stored in a desiccator. WB was chosen as the sole carbon source due to its high polysaccharide concentration (45% cellulose and 29% hemicellulose) in order to induce the enzymatic biosynthesis.^27^

The fungal preinoculum was prepared in Petri dishes of PDA and incubated for 7 days at 28 °C. SSF was performed in 250 mL Erlenmeyer flasks, containing 5 g of WB, supplemented with 5 mL of sterile nutrient solution (containing 0.35% (NH_4_)_2_SO_4_, 0.3% KH_2_PO_4_, 0.05% Mg_2_SO_4_·7H_2_O, and 0.05% CaCl_2_), and autoclaved (121 °C, 1 atm). Then, six mycelial disks (6 mm in diameter) were added to each flask, and the volume solution was completed to 20 mL with the nutrient solution to achieve 65% of moisture. The SSF assays were performed for 7 days at 28 °C in triplicate, using a total of 21 flasks (three flasks × seven time points).^21^ For the enzyme extraction, every 24 h, three flasks were taken from the incubator, added with 50 mL of distilled water, and submitted to orbital agitation at 150 rpm for 60 min. Afterward, the material was filtered using a nylon membrane and centrifuged for 10 min at 10,000 rpm (8760 g). The supernatant containing the crude enzymatic extract was aliquoted at – 20 °C.

### 2.3 Enzymatic Activities

Endoglucanase (CMCase), exoglucanase (avicelase), and xylanase were measured by the 3.5 dinitrosalicylic acid (DNS) method.^28^ The xylanase activity was determined using 10 μL of crude enzymatic extract and 90 μL of sodium acetate buffer (0.05 mol/L, pH 4.8) containing 1% (w/v) xylan (Sigma) as the substrate at 50 °C for 10 min. The reaction was stopped by the addition of 100 μL of DNS and boiled in a boiling water bath for 10 min, followed by cooling in an ice bath, and 800 μL of distilled water was added. The released reducing sugars were measured by spectroscopy at 540 nm. An enzymatic activity unit was defined as the quantity of enzyme required to liberate 1 μmol of β-d-xylose per minute. Exoglucanase and endoglucanase activities were quantified by the same methodology, with 1.0% Avicel or carboxymethylcellulose (CMC; Sigma) as substrates, respectively.^16^ One unit of enzymatic activity was defined as the amount of enzyme required to release 1 μmol of glucose per minute of reaction.

The β-glucosidase activity was quantified using 4 mmol/L p-nitrophenyl-β-d-glucopyranoside (PNPG, Sigma) as the substrate in a sodium citrate buffer solution (0.05 mol/L, pH 4.8) at 50 °C for 10 min.^16^ The reaction was stopped with the addition of 2 mL of 2 M sodium carbonate (Na_2_CO_3_). The released *p*-nitrophenol (pNP) was measured at 410 nm, and one enzymatic activity unit was determined as the amount of enzyme required to release 1 μmol of pNP per minute. The β-xylosidase activity was determined by the same method but using 4 mmol/L p-nitrophenyl-β-d-xylopyranoside (PNPX, Sigma) as a substrate.

### 2.4 β-Glucosidase Characterization

The physicochemical characteristics of the β-glucosidase from *T. atroviride* HTM were evaluated.^16^ The optimum pH was determined through the incubation of 50 μL of the enzymatic extract in 250 μL of PNPG (4 mmol/L), with variable buffer pHs from 3.5 to 8.0 at 50 °C for 10 min. The buffer solutions were 0.05 mol/L sodium citrate (from 3.5 to 7.0) and 0.05 mol/L sodium phosphate (7.5 and 8.0). The assays were stopped by adding 2 mL of 2 M Na_2_CO_3_. The optimum temperature was evaluated by incubating 50 μL of enzymatic extract at the optimum pH, at variable temperatures (30–80 °C), under the same experimental conditions. The pH stability was determined by incubation of the enzymatic solution for 24 h at room temperature in variable pH buffers from 3.5 to 8.0. Then, the residual β-glucosidase activity was measured under the respective optimum pH and temperature. For thermostability determination, the enzymatic extract was incubated at different temperatures (from 30 to 80 °C) for 1 h. The remaining enzymatic activity was evaluated at the optimum pH and temperature. All of the assays were carried out in triplicate.

### 2.5 Alkaline Pretreatment of Corn Wastes

Raw corn straw (RCS) and raw corn cob (RCC) samples, provided by JC Rações e Insumos Siderúrgicos (Uberlândia, MG, Brazil), were subjected to pretreatment with a 1% (w/v) sodium hydroxide (NaOH) solution for 1 h at 160 °C, as described in a previous study from our research group.^1^ Both raw and pretreated samples (PTCS and PTCC) were characterized for Klason lignin, cellulose, hemicellulose contents, and delignification rate, following the TAPPI T 222 om-88 and NREL LAP-002 methodologies. The chemical composition of pretreated samples, as performed in a previous study of our research group, demonstrated high efficiency in lignin removal and cellulose exposure (Table 1).^29^

**Table 1:** Chemical Composition (%) and Delignification Rate (%) in Raw (RCC) and Pretreated Corn Cob (PTCC) and Raw (RCS) and Pretreated Corn Stover (PTCS)^a^

biomass	lignin	cellulose	hemicellulose	delignification
RCC	15.59 ± 0.45	33.12 ± 0.56	35.15 ± 0.55	
PTCC	9.72 ± 0.50	53.02 ± 0.99	43.08 ± 0.73	72.19 ± 0.65
RCS	21.77 ± 0.37	31.97 ± 0.65	28.86 ± 0.37	
PTCS	12.30 ± 0.51	64.99 ± 0.86	26.47 ± 0.65	67.01 ± 0.74

a Adapted with permission from Fornazier et al.^1^

### 2.6 Effect of the Enzyme Loading on the Hydrolysis

Saccharifications were carried out in 50 mL Erlenmeyer flasks containing sodium citrate buffer (0.05 mol/L, pH 4.8) at a liquid-to-solid ratio of 10 mL/g (10% w/v), at 150 rpm and 50 °C for 72 h, in triplicate.^1^ The enzyme loading of 30 CBU (cellobiase/β-glucosidase unit) from the *T. atroviride* HTM cocktail was evaluated under two conditions: (I) the enzyme concentration per gram of total biomass (considering all the main components: lignin and polysaccharides) and (II) enzyme load per gram of cellulose in the material.^30^

The liquid fractions containing the free sugars (hydrolysates) were filtered through a 0.20 μm membrane (Chromafil Xtra CA-20/25) and centrifuged at 10,000 rpm for 10 min. Then, the total reducing sugar (TRS) release (g/L) was determined by the DNS method, using 100 μL of each hydrolysate and 100 μL of DNS, with the mixture heated at 100 °C for 10 min. Afterward, 800 μL of distilled water was added to the reaction, and the absorbance was measured using a UV–vis spectrophotometer (Metash, model UV-5100) at 540 nm.^28^

### 2.7 Fed-Batch Hydrolysis with Surfactant Addition

The fed-batch saccharification was carried out using the biomass and the enzyme load selected in Section 2.6, under the same conditions. The assay started with 10% substrate, and successive additions of 5% were carried out every 12 h until reaching 35% (w/v) of total solids and maintained up to 72 h.^1^ Afterward, the TRS were determined by the DNS method.^28^ Following this step, the effect of surfactant was evaluated using 1% (w/v) Tween 20 under the same conditions. Then, the TRS concentration was calculated.^28^

The presence of glucose, xylose, cellobiose, arabinose, and inhibitory compounds in the hydrolysates (acetic acid, formic acid, furfural—FF, and 5-hydroxymethylfurfural—HMF) was quantified by high-performance liquid chromatography (HPLC). Samples were diluted with the mobile phase (aqueous phosphoric acid solution—H_3_PO_4_, 0.1% v/v), filtered through a 0.20 μm membrane (Chromafil Xtra CA-20/25), and injected into a Shimadzu LC-20A Prominence chromatograph. The system employed a Supelcogel C-610H column at 32 °C, with isocratic elution at a flow rate of 0.5 mL/min. UV detection at 210 nm, refractive index detection, and standards for glucose, xylose, cellobiose, arabinose, organic acids, FF, and HMF were used.^1^ Enzymatic conversions (CE) were calculated according to eqs 1 and 2:^4^

$$\mathrm{CE}{\%}_{\mathrm{glu}\cos e}=\left(\frac{m_{\mathrm{glu}\cos e}\;f_{\mathrm{hg}}}{m_{\mathrm{sample}}\;y_{\mathrm{ic}}}\right)\times 100$$ 1

$$\mathrm{CE}{\%}_{\mathrm{xilose}}=\left(\frac{m_{\mathrm{xilose}}\;f_{\mathrm{hx}}}{m_{\mathrm{sample}}\;y_{\mathrm{ix}}}\right)\times 100$$ 2

where *m*_glucose_ and *m*_xylose_ (g) are the weights of glucose and xylose released by the enzymatic hydrolysis of glucan (cellulose) and xylan (hemicelluloses), respectively, *m*_sample_ (g) is the sample weight used in the enzymatic hydrolysis, *f*_hg_ = 0.9 and *f*_hx_ = 0.88 are the conversion factors, which take into account that one water molecule was added during the cellobiose and xylobiose hydrolysis, respectively, and *y*_ic_ and *y*_ix_ (%) represent contents of glucan and xylan in the substrate before the enzymatic hydrolysis, respectively.

### 2.8 Alcoholic Fermentation

In this stage, only the hydrolysate with the highest TRS and glucose concentrations, determined in Section 2.7, was used. Initially, the commercial yeast *Saccharomyces cerevisiae* Y-904 (Fleischmann) was precultivated in 5 mL of sterile YPD medium (1% yeast extract, 2% peptone, and 2% glucose) under aerobic conditions in a rotary shaker at 28 °C and 180 rpm for 24 h.^31^ Then, the hydrolysate was sterilized by autoclaving (0.5 atm), transferred to 50 mL Erlenmeyer flasks, and inoculated with 5% (v/v) of the yeast preinoculum. Fermentation was performed statically at 28 °C for 48 h, in triplicate.^1^ The fermented samples were centrifuged and filtered for the quantification of residual glucose and ethanol by HPLC.^4^ The ethanol productivity and yield were calculated based on the initial glucose and final ethanol concentrations, according to eqs 3 and 4:^20^

$$Q_{P}=\frac{\mathrm{Ethanol}}{t}$$ 3

$$Y_{P/S}=\left[\frac{\left[\mathrm{Ethanol}\right]}{0,511\times [\mathrm{Glu}\cos e]}\right]\times 100\%$$ 4

where *Q*_P_ represents the ethanol productivity (g/L h), Ethanol denotes the concentration of produced ethanol (g/L), *t* is the fermentation time (h), *Y*_P/S_ represents ethanol yield (%), and Glucose is the initial glucose concentration (g/L).

## 3 Results and Discussion

### 3.1 Enzyme Production

After SSF, the enzymatic activities from the *T. atroviride* HTM extract were measured, and the time course production curves were plotted. Among the cellulases, endoglucanase (CMCase) and exoglucanase (avicelase) exhibited moderate production, with maximum activities on the second day (18.85 and 44.27 U/g, respectively). Conversely, substantial biosynthesis (374.91 U/g) of β-glucosidase was observed at the seventh day of cultivation (Figure 1A).

**Figure 1:**
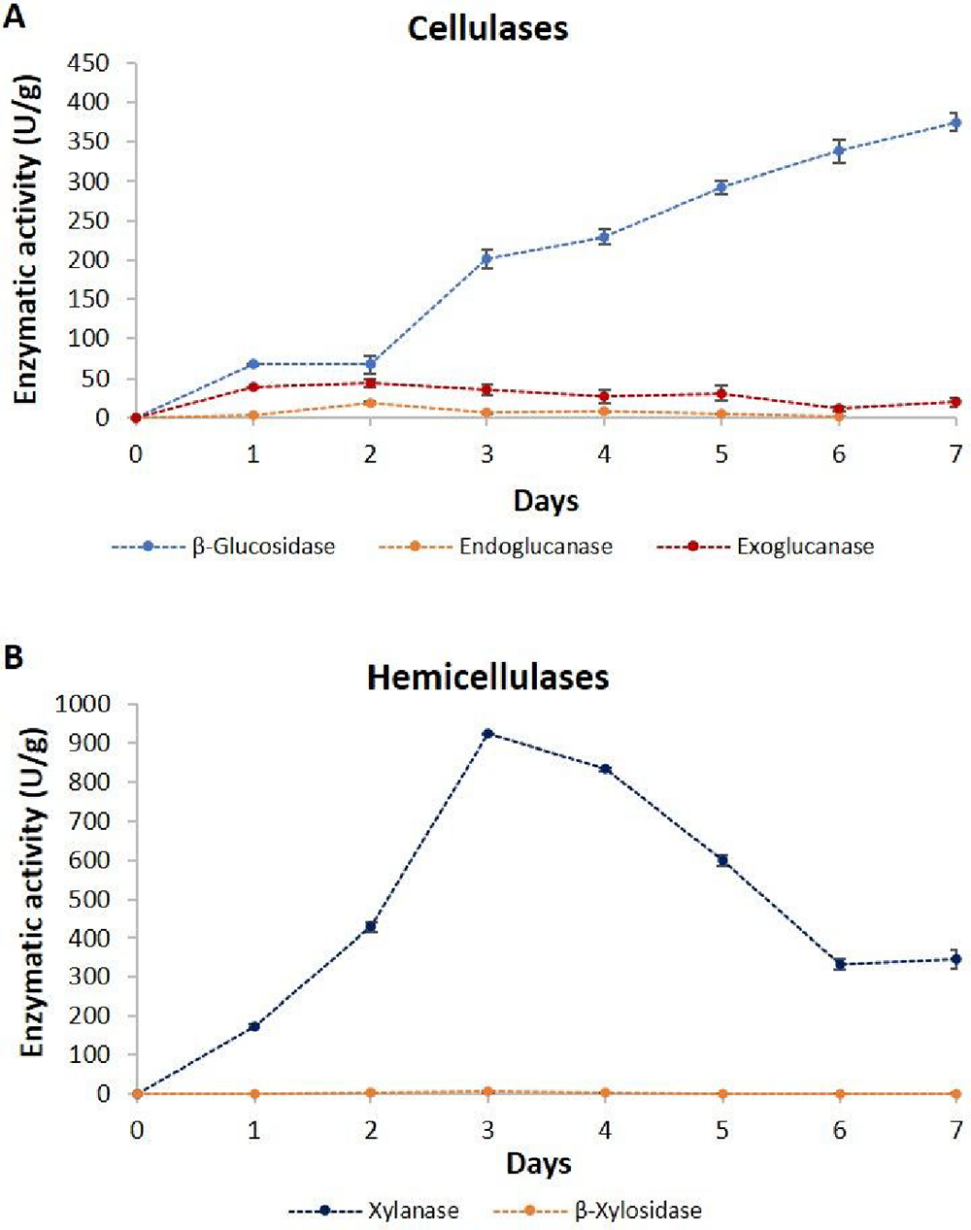
Production curves of cellulases (A) and hemicellulases (B) by *T. atroviride* HTM in SSF, using wheat bran (WB) as a substrate.

This notable production of β-glucosidase was superior to previous reports involving the genus *Trichoderma* ^16,27,35,40^ and can be due to the high protein content in WB. The WB used in this study contained 15.8% of protein,^27^ in accordance with the literature, in which the protein percentages in this material can vary between 12.0–18.0%.^32,33^ Besides, this substrate is rich in cellulose and has a large surface area, being an excellent inducer of this enzyme, without the necessity of supplementation.^27,34^ Previous studies have reported the inductive role of WB in the β-glucosidase biosynthesis by other fungal species during SSF. For example, Teles et al.^35^ carried out SSF with an *A. niger* 3T5B8 mutant strain and obtained the highest β-glucosidase activity (125.00 U/gds) in the medium containing WB as the sole carbon source.

This result is also in agreement with earlier studies reporting that longer incubation periods favor the β-glucosidase synthesis.^16,27^ For example, Corrêa et al.^27^ and Rodrigues et al.^16^ obtained peaks of β-glucosidase (33 and 46 U/g, respectively) both at the seventh day of cultivation of *T. asperellum*, using WB and sugar cane bagasse (SCB) as substrates. This behavior can be due to the synergism among the cellulases, wherein β-glucosidase plays a key role in the final cellulose conversion by hydrolyzing the β-1,4-glucosidic linkage of cellobiose, previously released by the other cellulases, into glucose, being a decisive factor for the saccharification effectiveness.^6,7^

Regarding the hemicellulases, the maximum xylanase activity was reached on the third day (920 U/g). Then, a gradual decrease was observed, a performance also observed by xylanases from other fungal species. For example, Rodrigues et al.^16^ reported a xylanase peak at the third day (785.5 U/g) by *T. asperellum* grown on SCB and WB (1:1). This high xylanase production is particularly important for saccharifications since the hemicelluloses can hinder the accessibility of cellulases to the cellulosic fraction. In this sense, these enzymes act mainly on the xylan chains, exposing the cellulose to the cellulase attack. Hence, the simultaneous production of cellulases and xylanase is highly relevant to maximize bioconversions.^36^ With respect to β-xylosidase, notable activities were not observed under the evaluated conditions.

The genus *Trichoderma* is well known as a good producer of cellulolytic and hemicellulolytic enzymes, with highlights for the species *T. reesei*, *T. harzianum*, and *T. longiflorum*, being *T. reesei* the most commonly used in industrial applications.^37,38^ However, there are few reports on the biosynthesis of such enzymes by *T. atroviride* under SSF and their further use in saccharifications.^19,39^ For example, Al-Qassab et al.^40^ investigated the synthesis of (hemi)­cellulolytic enzymes, employing *T. reesei* RUT-C30 and oil palm mesocarp fiber through SSF and obtained xylanase and β-glucosidase peaks of 1346.75 and 9.89 IU/gds, respectively. Rodrigues et al.^16^ cultivated the species *T. asperellum* in SCB and WB and obtained 785.5 U/g xylanase and 45.26 U/g β-glucosidase.

Comparing the present data with prior studies, particularly carried out with *T. atroviride*, the endoglucanase production at the initial stage of SSF was also observed by Bulgari et al.,^41^ who reported 83.3 U/g on the third day, using agricultural digestate and food waste as substrates. The enzymatic values from the current study were superior to Kovács et al.,^15^ who obtained 40.1 U/g xylanase using SCB as the substrate in SSF.

The present enzymatic profile was also higher than that obtained by Vaidyanathan et al.,^42^ who cultivated a genetically engineered *T. atroviride* strain in lignocellulosic biorefinery sludge and observed 84 U/g xylanase and 21 U/g cellulase. Our data were also greater than those found by Grujić et al.,^36^ who cultivated *T. atroviride* in spent mushroom compost and reported maximum xylanase and β-glucosidase of 2.31 and 2.0 U/mL, respectively. The results were also superior to Saldarriaga-Hernandez et al.,^19^ who evaluated the enzyme production by *T. atroviride* using brewer’s spent grains as the substrate, with maximum activities of β-glucosidase of 0.055 U/mL and xylanase of 0.125 U/mL over 15 days of SSF.

These comparisons demonstrate that the kind of substrate, the fungal strain, the nutritional profile of the medium, and the incubation time are determining factors for the enzymatic expression. Thus, the adopted strategy was effective not only due to the high enzyme concentrations but also due to the use of a low-cost substrate, such as WB. Moreover, the observed production profile favored both the rapid biosynthesis of xylanase and the progressive accumulation of β-glucosidase, combined with good levels of endoglucanase and exoglucanase. This flexibility allows the local production to be adjusted as needed, constituting a competitive advantage for bioprocesses based on lignocellulosic biomasses.

### 3.2 *T. atroviride* β-Glucosidase Characterization

Due to its key role in saccharifications, the β-glucosidase from *T. atroviride* HTM was characterized with respect to the pH and temperature effects. This analysis revealed an optimal pH around 4.5–5.0 (Figure 2a). This preference for slightly acidic pH is particularly advantageous since lignocellulose saccharifications generally occur in pH ranges between 4.5 and 5.5.^1,7^ This result is in accordance with previous studies, which have reported acidic optimal pHs for β-glucosidases.^7^ For example, Kovács et al.^15^ also observed an optimal pH of 5.0 for β-glucosidases from the *Trichoderma* genus cultivated in other carbon sources. The evaluation of pH stability (Figure 2b) confirmed the suitable performance of this β-glucosidase under acidic conditions, maintaining more than 70% of its initial activity within this operational range.^1,4^

**Figure 2:**
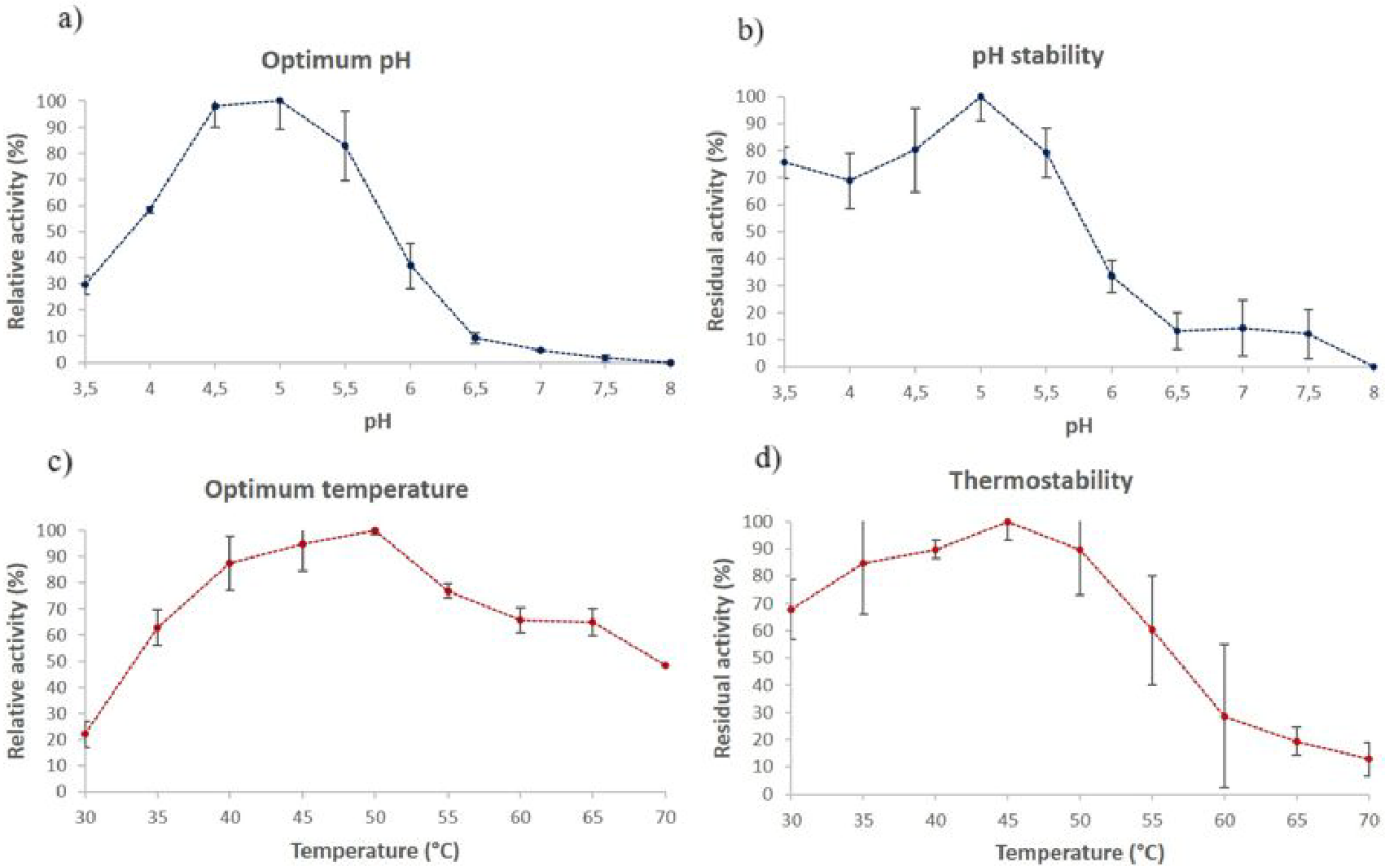
Effects of pH and temperature on the β-glucosidase activity from *T. atroviride* HTM cocktail: (a) optimum pH, (b) optimum temperature, (c) pH stability, and (d) thermostability.

The optimal temperature was achieved around 45–50 °C (Figure 2c), which is also appropriate for application in biomass hydrolysis, which typically operate at 50 °C.^16,43^ Besides, it showed thermostability up to 50 °C, retaining >80% of its initial activity after 1 h of incubation (Figure 2d). However, above 55 °C, the activity considerably decreased, a typical profile for β-glucosidases from other fungal sources.^6,16,27^ Therefore, this new β-glucosidase presented high activity and stability under acidic pH and moderate temperatures, which are compatible and desirable for bioconversions, highlighting the potential of the *T. atroviride* HTM cocktail.

### 3.3 Hydrolysis with the Enzyme Load Based on the Concentration of Cellulose or Biomass

Raw and pretreated CC and CS were submitted to saccharifications with 10% solids and enzyme load per gram of cellulose or biomass. The results showed that the pretreated hydrolysates exhibited TRS concentrations higher than those of the raw samples, indicating the effectiveness of the alkaline pretreatment in the improvement of the hydrolytic effectiveness (Figure 3). In PTCC, increases of 183% (30 CBU/g cellulose) and 57% (30 CBU/g biomass) were observed in comparison to RCC. Likewise, PTCS recorded increments of 280% (30 CBU/g cellulose) and 254% (30 CBU/g biomass) when compared to RCS. The highest TRS release was obtained in the hydrolysis of PTCC using the enzyme loading per gram of cellulose (8.97 g/L). These data are in agreement with previous studies which have demonstrated the effectiveness of alkaline pretreatment in the enhancement of saccharifications,^1,44^ mainly due to the high removal of lignin and the preservation of cellulose.^45,46^ Since the presence of lignin restricts the accessibility of enzymes to the polysaccharides, the observed high delignification (Table 1) was crucial to reduce the degree of polymerization and increase the porosity, making the cellulose more accessible for the enzymatic attack in the hydrolysis stage.^1,4^

**Figure 3:**
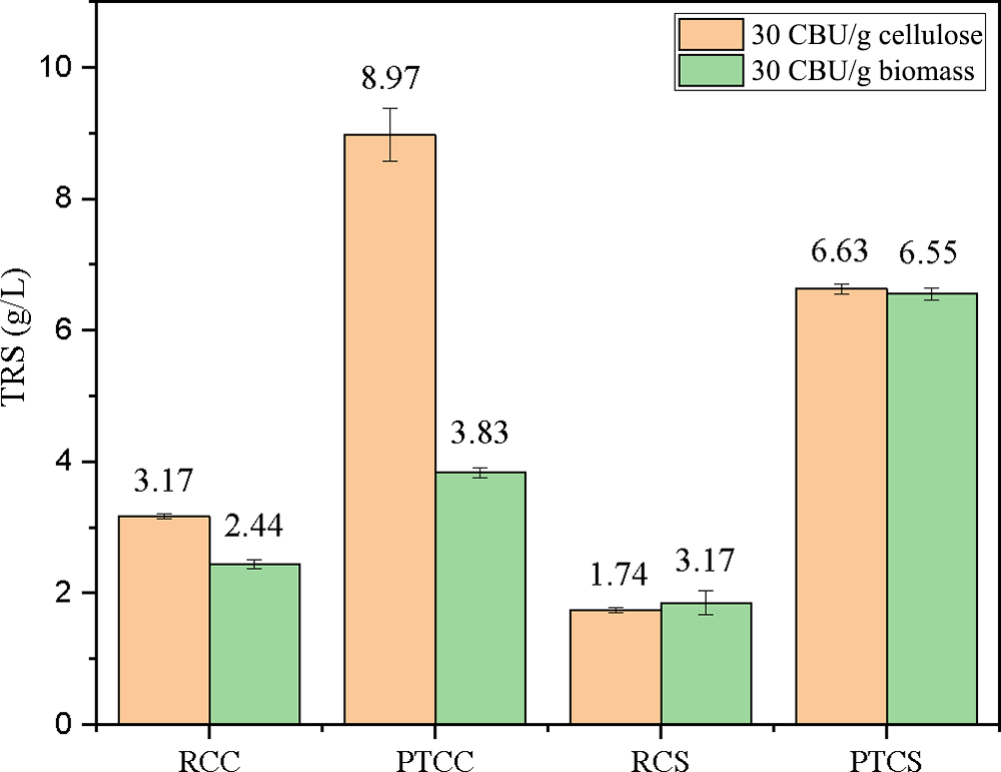
Total reducing sugar (TRS) contents in raw corn cob (RCC) and pretreated corn cob (PTCC), and raw corn straw (RCS) and pretreated corn straw (PTCS) after the enzymatic hydrolysis carried out with 10% substrate and 30 CBU of *T. atroviride* HTM cocktail, added per gram of cellulose or biomass.

Besides, the achieved TRS values were consistent with the expected performance for hydrolysis carried out with locally produced cocktails.^16^

Considering saccharifications particularly carried out with *Trichoderma* enzymatic solutions, these results are superior to those obtained by He et al.,^47^ who conducted hydrolysis using a *T. reesei* enzyme complex (7 FPU/g dry substrate and 1% solid load) and observed 4.67 g/L TRS in PTCC, corresponding to an increase of 46.7% in comparison to RCC. Similarly, Chakraborty et al.^48^ used a crude *Trichoderma*sp*.* RCK65 cocktail produced by SSF, composed of 146.53 U/g CMCase, 39.07 U/g FPase, and 105.02 U/g β-glucosidase, to saccharify chlorite-pretreated *Prosopis juliflora* (a woody substrate), using 20 FPase/g and 1% Tween 80, and resulted in 83% (w/w) conversion.

Thus, these data emphasize the importance of considering both the chemical composition of the biomass and the enzymatic profile of the cocktail to adequately adjust biomass utilization and improve sugar yields. Based on these results, hydrolysis optimization was continued only for PTCC, with the enzyme load adjusted per gram of cellulose.

### 3.4 Fed-Batch Hydrolysis with Surfactant Addition

The fed-batch saccharification of PTCC without surfactant achieved 15.86 g/L TRS at 35% solids, representing an improvement of 76.8% in comparison to conventional hydrolysis (Figure 4). This result can be due to the gradual substrate addition, which decreases the negative effects of high solid loadings.^49^

**Figure 4:**
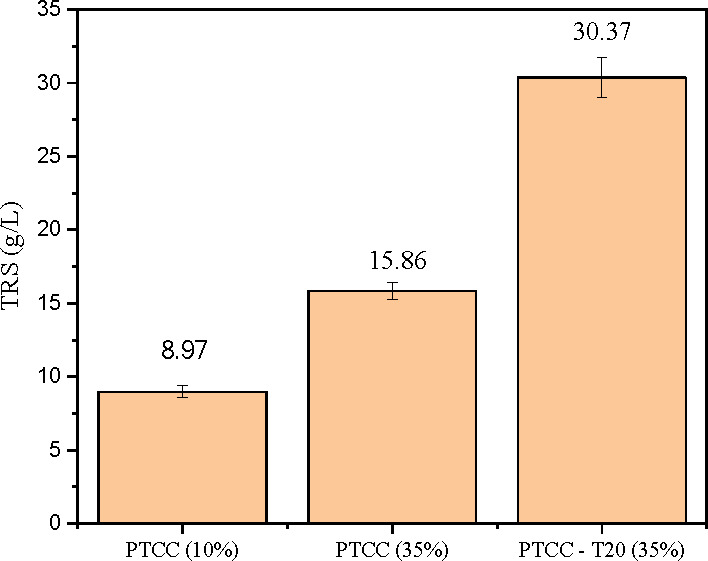
Fed-batch hydrolysis of pretreated corn cob (PTCC) combined with the addition of 1% (w/v) Tween 20, using 30 CBU of *T. atroviride*HTM cocktail per gram of cellulose. T20: Tween 20.

Some authors have highlighted that this strategy prevents abrupt increases in the viscosity, mass transfer limitations, formation of concentration gradients, and nonspecific adsorption of enzymes onto residual lignin.^20,50,51^ This way, it collaborates to maintain the fluidity of the reactional system, facilitating the enzyme–substrate contact and enhancing the bioconversion.^52^

Additionally, the use of 1% Tween 20 significantly enhanced the TRS release in PTCC, with a production of 30.37 g/L, corresponding to an increase of 91,48%, in comparison to the PTCC fed-batch hydrolysis without the surfactant. This noteworthy improvement can be explained by the surfactant ability to reduce the interfacial tension between the solid and liquid phases, as well as modify the interactions between enzymes and lignin.^49^ Thus, the use of Tween 20 can be an auxiliary tool to overcome limitations associated with high-solid processing. These effects contribute to further decreasing the nonproductive enzyme adsorption, increasing the catalytic stability and improving the cellulose accessibility.^53^ As evidenced by Wang et al.,^54^ these surfactants initially bind to chemical groups released from lignin, facilitating the enzymatic access to cellulose. This effect was corroborated by Lee et al.,^55^ who emphasized that the Tween 20 acts by blocking hydrophobic binding sites on the lignin surface, contributing to the reduction of adsorption and preserving the catalytic sites. Seo et al.^23^ also reported that Tween 20 interacts with lignin through its hydrophobic tails, while its hydrophilic heads remain oriented toward the aqueous phase, forming a layer that blocks the enzyme adsorption sites. This blockage contributes to an increased fraction of enzymes available to act on the target polysaccharides. Furthermore, this surfactant presents compatibility with hydrolytic enzymes and may enhance their stability.^55^ Narra et al.^56^ also evidenced the remarkable role of fed-batch hydrolysis coupled with the use of surfactants. These authors saccharified NaOH-pretreated rice straw, using 25% (w/v) biomass, 1% Tween 80, and 9 FPU per gram of substrate of an on-site enzyme preparation from *A. terreus*, obtaining a hydrolytic efficiency of 76%.

The fed-batch hydrolysis with 35% PTCC and 1% Tween 20 was also reflected in higher monosaccharide contents (Table 2). In these conditions, the glucose and xylose concentrations increased 127 and 123%, respectively, in comparison to the hydrolysis without surfactant. Cellobiose also showed a significant increase of 115%, while arabinose exhibited an increase of 225%.

**Table 2:** Monosaccharides and Cellobiose Concentrations (g/L) in the Pretreated Corn Cob (PTCC) Hydrolysates after Fed-Batch Saccharifications Carried Out with 35% Solids and 1% Tween 20 (T20)

biomass	glucose	xylose	cellobiose	arabinose
PTCC	3.21 ± 0.73	4.45 ± 0.96	4.01 ± 0.91	0.12 ± 0.08
PTCC – T20	7.28 ± 0.93	9.93 ± 0.91	8.65 ± 0.59	0.39 ± 0.03

These data reinforce the positive role of Tween 20 in the fermentable sugar release, probably due to its ability to reduce nonproductive adsorption while simultaneously increasing the polysaccharides access.^53,57^ Thus, the adopted strategy proved to be promising not only due to its improved efficiency but also because of its operational simplicity and low cost, features that are highly desirable for biorefineries.^57^

The obtained glucose concentration surpasses that reported by Kovács et al.,^58^ who carried out hydrolysis of steam-pretreated wood using a *T. atroviride* cocktail produced by submerged fermentation. In that study, the TUB F-1663 cocktail was applied in reactions with 20 g/L solids, resulting in 4.9 g/L glucose. These differences can be attributed to some factors, such as the kind of pretreatment, the kind of biomass, and the fungal strain, but mainly attributed to the high solid loading and the application of surfactant, which reduced the enzyme losses. Additionally, SSF demonstrated a greater capacity to induce β-glucosidase and xylanase production, resulting in a more balanced enzymatic cocktail.

Another relevant factor that may impact the hydrolytic performance is the presence of inhibitors. Furan derivatives and organic acids can be generated and negatively affect both the hydrolysis and alcoholic fermentation.^1,59^ Among them, acetic and formic acids, FF, and HMF, derived from the degradation of pentoses and hexoses, are highlighted by their toxic effects.^59^ However, in the present study, FF and HMF were not detected under the evaluated conditions (Table 3). Moreover, the concentrations of acetic and formic acids were low, with maximum values of 0.45 and 0.25 g/L, respectively.

**Table 3:** Concentrations (g/L) of Organic Acids, HMF, and FF in Pretreated Corn Cob (PTCC) Hydrolysates after Fed-Batch Hydrolysis Performed with 35% (w/v) of Solids^a^

biomass	formic acid	acetic acid	HMF	FF
PTCC	0.25 ± 0.13	0.45 ± 0.34	ND^b^	ND
PTCC - T20	0.19 ± 0.11	0.31 ± 0.18	ND	ND

a T20: Tween 20; FF: furfural; HMF: 5-hydroxymethylfurfural.

b ND = not detected.

Thus, the absence of FF compounds and the very low levels of organic acids may have contributed to the stability of the enzymatic system during hydrolysis, even under high solid loads. Moreover, a marked decrease in the concentration of these acids was observed in the presence of Tween 20 (PTCC-T20), which may have positively contributed to the increase in the release of fermentable sugars.

Therefore, these data demonstrated the potential of the homemade *T. atroviride* HTM extract in converting corn cob into fermentable sugars and reinforced the effectiveness of the combined strategy of fed-batch hydrolysis with the surfactant use.

### 3.5 Alcoholic Fermentation

The fermentation of PTCC hydrolysate from the fed-batch saccharification in the presence of Tween 20 produced 3.03 g/L ethanol, with 88.66% glucose consumption and 83.48% theoretical conversion yield (YP/S). These data are in accordance with previous studies about 2G ethanol production from saccharifications carried out with on-site enzyme cocktails. For example, Rodrigues et al.^20^ conducted a fed-batch hydrolysis of hydrothermally pretreated SCB, with a final solid loading of 30% (w/v) and 5% Triton X-100, employing an in-house cocktail from a consortium among *A. niger*, *T. versicolor*, and *P. ostreatus*. The fermentation of the hydrolysate resulted in an ethanol productivity of 0.035 g/L·h and a theoretical yield of 88.03%, respectively.^20^

This elevated glucose conversion into ethanol indicated the viability of this new homemade *T. atroviride* cocktail in bioconversions. Moreover, the absence of FF and HMF and the highly reduced concentrations of organic acids reinforce the effectiveness of the adopted strategy of combining fed-batch hydrolysis and the use of the surfactant. These acids can cause intracellular acidification and increase the energy consumption to maintain the pH, compromising the fermentative efficiency,^59^ with tolerable maximum concentrations of up to 3.0 g/L^60^ for acetic acid and 2 g/L for formic acid.^61^ Thus, their decreased concentrations could have reduced the negative impact on fermentation. Therefore, these results reflected the notable positive effect of the addition of surfactants to minimize the inhibitor formation, favoring the release of fermentable sugars and, consequently, the ethanol production.

## 4 Conclusions

The present study demonstrated the feasibility of local production of a novel *T. atroviride* enzymatic solution by SSF, with elevated β-glucosidase and xylanase activities. The β-glucosidase from this cocktail revealed high activity and stability in pH and temperature ranges compatible with the optimal hydrolysis conditions. The application of this cocktail in the fed-batch saccharification of alkali-pretreated corn cob resulted in significant TRS release. The use of 1% Tween 20 notably contributed to the increase of the levels of fermentable sugars and the reduction of the formation of inhibitors. Alcoholic fermentation of the PTCC hydrolysate showed high glucose consumption (88.66%) and an elevated theoretical conversion yield (83.48%). These data evidence the importance of exploring new endophytic fungal strains for the sustainable biosynthesis of enzymatic cocktails and their use in saccharifications, contributing to the advancement of 2G biofuel production and the circular economy.
